# *Salvia miltiorrhiza* polysaccharide-Mn²⁺-PLGA as an adjuvant in H9N2 vaccine: Immunomodulatory effects in chickens

**DOI:** 10.1016/j.psj.2025.105599

**Published:** 2025-07-26

**Authors:** Yixuan Zhu, Pengfei Gu, Yongzhan Bao, Bowen Song, Jinglu Zhang, Xiao Wang, Wanyu Shi

**Affiliations:** a College of Traditional Chinese Veterinary Medicine, Hebei Agricultural University, No. 2596 Lekai South Street, Baoding 071000, China; b Hebei Key Laboratory of Traditional Chinese Veterinary Medicine, Baoding, 071001, China

**Keywords:** H9N2, *Salvia miltiorrhiza* polysaccharide, Mn^2+^, PLGA, Adjuvant

## Abstract

H9N2 is an increasing threat to global poultry production and human health. Vaccination of poultry is a key element of disease control in endemic countries, but the inactivated H9N2 vaccine needs to be used with adjuvants to compensate for its weak immunogenicity. *Salvia miltiorrhiza* polysaccharide (SMP) and Mn^2+^ have received extensive attention because of their ability to enhance adaptive immunity and stimulate IFN-β secretion, respectively. Our previous studies had indicated that Mn^2+^/SMP encapsulated by PLGA (MS-PLGA) can be used as an effective adjuvant for the model antigen (ovalbumin), triggering a potent and durable immune response in mice. Here, we due to confirm the effect of MS-PLGA in chickens. we successfully prepared MS-PLGA-H9N2 and after immunization, it generated stronger specific IgG antibody and higher HI titers, and it also can activate CD4^+^ and CD8^+^*T* cells in peripheral blood and spleen. Moreover, both concentrations of the serum cytokine IFN-γ (Th1), IL-6 (Th2) and IFN-β are increased, demonstrating Th1/Th2 without bias. To elucidate the regulatory mechanism of MS-PLGA-H9N2, multi-omics analysis and immunofluorescence of the spleen were explored. The GO and KEGG pathways revealed that immune response and defense response were promoted and lymphocytes of spleen were activated. Overall, MS-PLGA-H9N2 has the potential to be clinically induce to induce potent immune responses in chickens.

## Introduction

Avian influenza viruses (AIVs) are broadly classified as highly pathogenic (HP) and low pathogenic (LP), causing significant annual economic losses to the global poultry industry year (França and Brown, 2014). H9N2 is the most prevalent LPAIV subtype circulating in poultry (Bo et al., 2021; Channa, et al., 2021; Le, et al., 2023). It is listed as a zoonotic influenza virus by the World Health Organization and a potential pandemic virus by the United States Centers for Disease Control and Prevention (CDC) (Alqazlan, et al., 2021). H9N2 has an error-prone RNA polymerase, which facilitates recombination, occasionally leading to the emergence of zoonotic reassortant strains, such as H7N9 and H10N8 (Li, et al., 2014; Wei, et al., 2016; Horman, et al., 2018). Therefore, limiting the prevalence of H9N2 through 'active prevention' is of great public health significance. In addition to strict biosafety measures, countries such as China, South Korea and Egypt have developed vaccination programs for H9N2 in poultry farms, using oil adjuvants with inactivated H9N2 vaccine (Peacock, et al., 2019). However, H9N2 outbreaks have been reported even in vaccinated flocks (Park, et al., 2011), probably due to the low antigenicity of current vaccines. However, the efficacy of vaccines depends not only on the antigen components but also on the adjuvants (Facciolà, et al., 2022; Pogostin and McHugh, 2021).

In recent years, researchers are exploring herbal polysaccharides as adjuvants in vaccines, because of immunomodulatory effect on the immune response and biosafety (Ren, et al., 2021; Zhang, et al., 2023; Zhao, et al., 2023; He, et al., 2024). Recently, the research on *Salvia miltiorrhiza* polysaccharide (SMP) mainly focused on anti-tumor, antioxidant, and alleviating liver or kidney damage (Liu, et al., 2013; Jiang, et al., 2015). Hence, we explored the adjuvant effect of SMP, so we first extracted and purified SMP from the dry root of *Salvia miltiorrhiza*. Secondly, we used SMP as an adjuvant for the model antigen ovalbumin (OVA), and observed that it can effectively enhance the humoral and cellular immune responses in mice (Zhu, et al., 2024). However, SMP also has some drawbacks, such as a short half-life, low bioavailability, and no antiviral activity.

Manganese (Mn) is one of the essential trace elements in the body and is necessary for various physiological processes. Current studies have shown that Mn^2+^ can be used as an agonist of the cGAS-STING pathway, which can effectively promote the release of interferon-β (IFN-β) and pro-inflammatory cytokines to trigger potent antiviral immunity (Lv, et al., 2020; Sun, et al., 2021; Zhang, et al., 2021). However, direct application of soluble Mn^2+^ has no local depot effect (Grimaldi, et al., 2017; Zhou, et al., 2023). In recent years, nano-delivery systems have been widely used in the field of adjuvants, such as chitosan and liposomes etc. (Grimaldi, Incoronato, Salvatore and Soricelli, 2017; Lung, et al., 2020). The sustained release of nano-delivery systems is better for solve the problems of SMP and Mn^2+^, and ensures that antigens and adjuvants can be taken up by the same APCs, avoiding the immune tolerance caused by a single antigen (Goldberg, 2015). Among various nanomaterials, we chose poly (lactic-co-glycolic acid) (PLGA), a material with high biosafety, to encapsulate Mn^2+^ and SMP (MS-PLGA), where MS-PLGA used as the OVA adjuvant could stimulate mice to produce potent innate and adaptive immune responses for a long time (Zhu, et al., 2025).

In this study, based on our previous study, we prepared the MS-PLGA-H9N2 vaccine delivery system and immunized chickens to evaluate whether MS-PLGA functions as an adjuvant or not in chickens by assessing its ability to induce innate and adaptive immune responses. Furthermore, through immunofluorescence, integrated transcriptomic and proteomic analysis of the spleen, we explored the underlying mechanisms of its immunomodulatory effects. Our findings provide insights into the potential of MS-PLGA as an adjuvant in inactivated H9N2 vaccine.

## Materials and methods

### Material

H9N2 inactivated antigen was purchased from Harbin Guosheng Biotechnology Co., Ltd. ISA-206 adjuvant was purchased from Sepco Special Chemicals Co., Ltd. (China). Alhydrogel adjuvant from InvivoGen (France). Chicken peripheral blood lymphocyte isolation solution was purchased from Solarbio Life Sciences (China). Mouse anti-chicken antibodies were purchased from Southern Biotech (USA).

### Experimental chicks

Hebei Dawu Agriculture and Animal Husbandry Group Co., Ltd. (Baoding, China) provided 90 a day old non-immunized Arbor Acres (AA) chickens. The temperature and humidity of the nursing room were adjusted at any time according to the age of chicks (21∼36°C; Humidity positive pressure 40 %∼60 %), allowing ad libitum feed and water. All animal experiments were carried out according to *the laboratory animal care and use guidelines*. The experiment has been approved by the Animal Care and Use Committee of Hebei Agricultural University (Confirmation number: 2022161).

### Preparation of MS-PLGA-H9N2 nanoparticles

MnCl_2_·4H_2_O, SMP and H9N2 were dissolved in 200 μL internal aqueous phase, and PLGA was dissolved in 2 mL dichloromethane to form the oil phase. The internal aqueous phase was added to the oil phase, and first emulsion was prepared by ultrasonic emulsification in the ice bath (60 W, 1 min). Then the first emulsion was added to the external aqueous phase containing F68, and the double emulsion was formed by ultrasonic emulsification in the ice bath (65 W, 5 min). The organic solvent was evaporated after stirring for 4 h to form a stable W1/O/W2 emulsion and named as MS-PLGA-H9N2.

### Characterization of MS-PLGA-H9N2

The average size, poly dispersity index (PDI) and zeta potential of MS-PLGA-H9N2 were analyzed by Dynamic Light Scattering (DLS) using a Malvern Zetasizer Nano instrument (*n* = 3) every week at 4°C to observe it stability. The morphology of MS-PLGA-H9N2 nanoparticles was observed by transmission electron microscopy (Talos L120C transmission electron microscope, voltage 120 KV). In order to determine the encapsulation efficiency of H9N2 in MS-PLGA, the nanoparticles vaccine was centrifuged at 12,000 rpm for 30 min, and the free H9N2 antigen was collected in the supernatant. The encapsulation efficiency of H9N2 in the nanoparticles was determined using the BCA protein concentration determination kit.

Adsorptionefficiency=(Totalantigen−Supernatantantigen)/Totalantigen%

### Immunization of chickens

Arbor Acres (AA) chickens were randomly divided into 6 different groups (*n* = 15): PBS control group (Control), inactivated H9N2 group (H9N2), Alhydrogel adjuvant-inactivated H9N2 mixed group (Algel-H9N2), ISA oil adjuvant-inactivated H9N2 mixed group (ISA-H9N2), MS-PLGA-inactivated H9N2 group, and unencapsulated MS-inactivated H9N2 (MS-H9N2). The concentration of inactivated H9N2 in each group was 0.75 mg/a chick. On the 9th day of age, 300 μL vaccine was subcutaneously injected into the neck of the chicks (Gu, et al., 2020), and the second immunization was performed at an interval of 14 days.

### Monitoring of body weight and antibody level

The body weight of chickens in each group was weighed every 7 days from 1 day old (*n* = 10). Serum samples were collected on the 21st, 28th and 35th days after the first immunization. The secretion of IgG antibodies was determined by H9N2 specific IgG double antigen sandwich method ELISA kit (*n* = 10). Hemagglutination inhibition (HI) titers of serum samples were determined by standard HI assays (*n* = 10) (Elaish, et al., 2019; Lee, et al., 2020).

### Peripheral blood lymphocyte activation

On the 21st day after first immunization, blood samples were collected from each group of immunized chickens (*n* = 5) and processed according to the instructions of the peripheral blood lymphocyte separation fluid for processing (Solarbio). Then, peripheral blood lymphocytes were stained with Anti-Chicken CD3-FITC, Anti-Chicken CD4-PE and Anti-Chicken CD8-APC, and analyzed by flow cytometry.

### Determination of serum cytokines

On the 21st day after first immunization, the serum of chickens in each group was collected (*n* = 10), and the levels of IFN-β, IL-6 and IFN-γ in serum were determined according to the instructions of the solid phase sandwich ELISA kit.

### Histological analysis and biosafety evaluation

The spleen, thymus and bursa of Fabricius (*n* = 3) of chicken were collected on the 21st and 35th day, and hematoxylin and eosin (HE) staining was performed to observe the histological changes of organs in each group. The serum biochemical index, including hepatic enzymes (AST, ALT, ALP), and renal parameter (LDH, BUN), were quantitatively analyzed using an automated biochemistry analyzer. The biological safety of each group of adjuvants was comprehensively evaluated.

### Combined analysis of spleen transcriptome and proteomics

On the 35th day after first immunization, the spleen of control, H9N2 and MS-PLGA-H9N2 groups were collected and frozen in liquid nitrogen (*n* = 6). Transcriptome and proteomics tests were performed by Novogene Co., Ltd.

### Immunofluorescence of tissue sections

On the 21st and 35th day, the spleen of control, H9N2 and MS-PLGA-H9N2 groups were collected and fixed in paraformaldehyde to make paraffin sections (*n* = 3). The number and expression of CD4^+^ and CD8^+^ *T* cells in the spleen were observed by immunofluorescence.

### Data analysis

All results are expressed as mean ±standard error (SEM). Duncan multiple range test was used to evaluate the statistical significance of the difference. Probability value (p) less than 0.05 was considered statistically significant.

## Results

### Characterization of MS-PLGA-H9N2

**Fig. 1:**
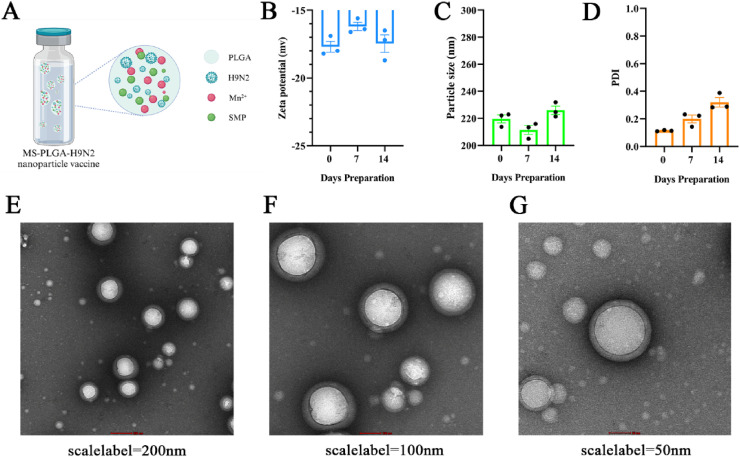
(A) The schematic illustration of MS-PLGA-H9N2 nanoparticles. (B), (C) and (D) The characteristics of MS-PLGA-H9N. (E), (F) and (G) Morphology of MS-PLGA-H9N2 under the TEM.

The pattern diagram of nanoparticle preparation was shown in Fig. 1A, in which the agonist SMP/Mn^2+^ and inactivated H9N2 antigen were encapsulated by PLGA. The average particle size of MS-PLGA-H9N2 was 219.6 ± 5.10 nm, the negative surface charge was 17.7 ± 0.7 mV, and the polydispersity index (PDI) was 0.114±0.005. Subsequently, MS-PLGA-H9N2 was stored at 4°C for 14 days, and it showed good stability (Fig. 1B–D). Transmission electron microscopy (TEM) images showed that MS-PLGA-H9N2 was a spherical particle with a smooth surface (Fig. 1E–G) and we determined the encapsulation efficiency of H9N2 by the BCA method to be 69.40 %.

### Body weight and antibody monitoring

**Fig. 2:**
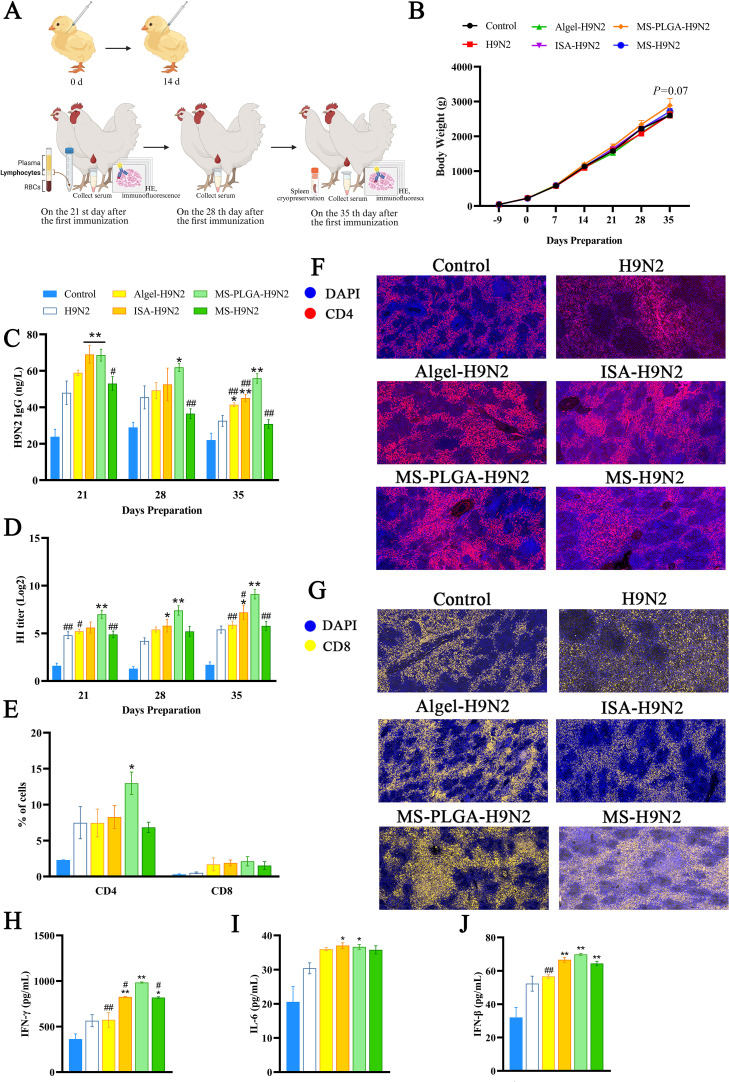
(A)Schematic diagram of the immunization protocol. (B) Effects of adjuvants on chicken body weight. (C) Dynamic changes in H9N2-specific IgG antibody titers. (D) Dynamic changes in HI titer. (E) Activation levels of CD4^+^ and CD8^+^*T* cells in peripheral blood lymphocytes. (F-G) Immunofluorescence of spleen tissue sections, 10 × 20, scale = 50 μm. (H-J) Serum cytokine levels of IFN-γ, IL-6 and IFN-β. * represents compared with H9N2, ^#^ represents compared with MS-PLGA-H9N2, *p<0.05*.

Chicks received their first immunization at 9 days of age, which was designated as day 0 in the immunization schedule (Fig. 2A). From −9 to 35 days, we weekly monitored the body weight of chickens across all experimental groups. None of the adjuvant formulations significantly affected overall growth performance. Notably, beginning on day 35, chickens in the MS-PLGA-H9N2 group exhibited greater body weight compared to the control group (Fig. 2B).

On the 21st day, ISA-H9N2 and MS-PLGA-H9N2 groups could significantly increase the titer of H9N2-specific IgG compared with H9N2 group. On the 28th day, the ISA-H9N2 group's antibody titer began to recede, while MS-PLGA-H9N2 mounted increased production of IgG antibodies. On the 35th day, the antibody titer of the H9N2 group decreased significantly (Fig. 2C).

Hemagglutinin (HA) is the main glycoprotein on the surface of the influenza virus, serves as a key target antigen for humoral immune responses (Zhang, et al., 2014). To evaluate HI titer, we performed hemagglutination inhibition (HI) assay, the results showed MS-PLGA-H9N2 could maintain HI titer at a high level on the 21st, 28th and 35th day (Fig. 2D), and the ISA-H9N2 also had a good stimulation effect on HI titer on the 28th and 35th day.

### Activation of peripheral blood lymphocyte and cytokine secretion

To further study the adjuvant potential of MS-PLGA-H9N2 nanoparticles, we analyzed CD4^+^ and CD8^+^*T* lymphocyte activation in peripheral blood lymphocytes by flow cytometry. On the 21st day, the MS-PLGA-H9N2 group significantly increased the percentage of CD3^+^CD4^+^ *T* and CD3^+^CD8^+^ *T* lymphocytes, and was higher than the other adjuvant groups (Fig. 2E). The splenic immunofluorescence sections on the same day showed the same results that MS-PLGA-H9N2 had a good activation effect on CD4^+^ and CD8^+^ *T* cells (Fig. 2FG). Furthermore, we detected the concentration of serum cytokines. Both ISA-H9N2 and MS-PLGA-H9N2 could significantly stimulate the secretion concentration of IFN-γ (Th1) and IL-6 (Th2), along with the antiviral cytokine IFN-β (Fig. 2HIJ).

### Biosafety evaluation

**Fig. 3:**
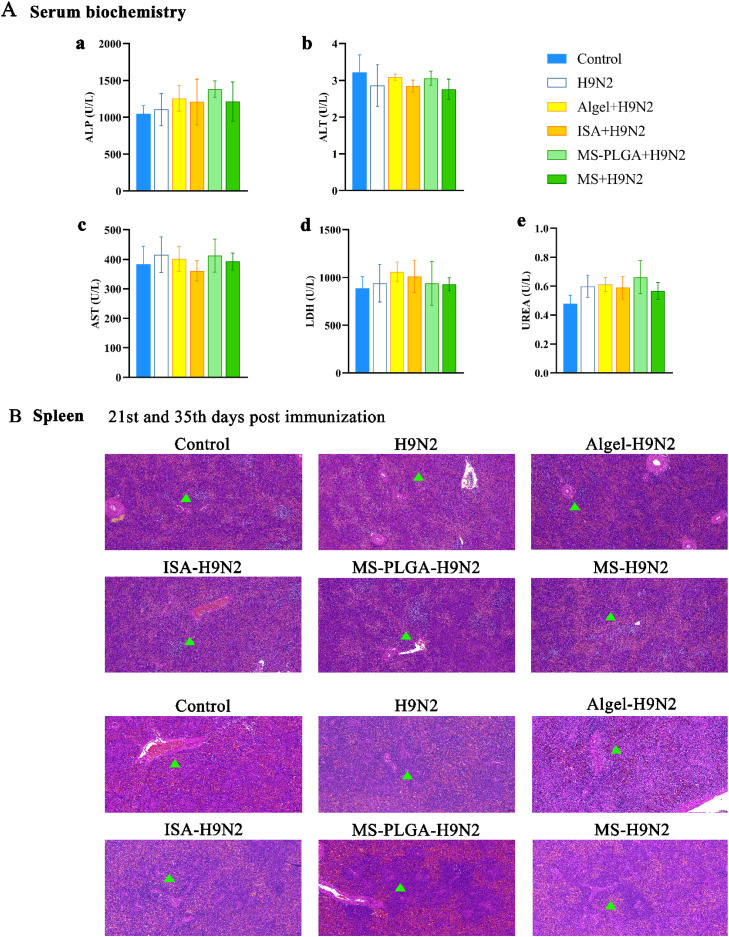
Biochemical and histological analyses. (A) Serum biochemical parameters. (B) Splenic tissue section, “▲” representing the splenic corpuscle and the area of the periarterial lymphatic sheath. 10×10, scale=100 μm.

After the first immunization, serum samples and immune organs were collected from chickens (*n* = 4). The results showed that there was no significant difference in biochemical indexes of liver and kidney between the adjuvant groups and the control group (Fig. 3A). No obvious tissue damage or inflammatory infiltration was observed in the spleen, thymus and bursa of Fabricius (BF) (Fig. 3B, Fig. S3, S4), indicating that the adjuvants had good biological safety. Moreover, we observed the change of organizational structure. The spleen was mainly composed of red pulp and white pulp. In the adjuvant group, the volume of splenic corpuscle was significantly increased, the splenic cord was widened, the number of lymphocytes was increased, and obvious periarterial lymphatic sheath was observed around the artery. The thymus consists of a chromophilic cortex and a hypochromatic medulla. Immature T lymphocytes are distributed in the cortex under the screening of cortical thymic epithelial cells (cTECs), subsequently, they migrate into the medulla, interacting with medullary thymic epithelial cells (mTECs) to undergo further screening and maturation before being released as functional T cells. It can be seen from Fig. S3 that the cortex and medulla are clearly demarcated, and the number of cells in the antigen and adjuvant groups is increased compared with the Control group. Bursa of Fabricius is a unique immune organ of birds. B cells proliferate and differentiate here. From Fig. S4, it can be seen that the size of lymphoid follicles is normal, the boundary between cortex and medulla is clear, and the volume of follicles at 35th day is larger than that at 21st day, showing an oval shape.

### Transcriptomic analysis showed MS-PLGA-H9N2 induces a potent immune response

**Fig. 4:**
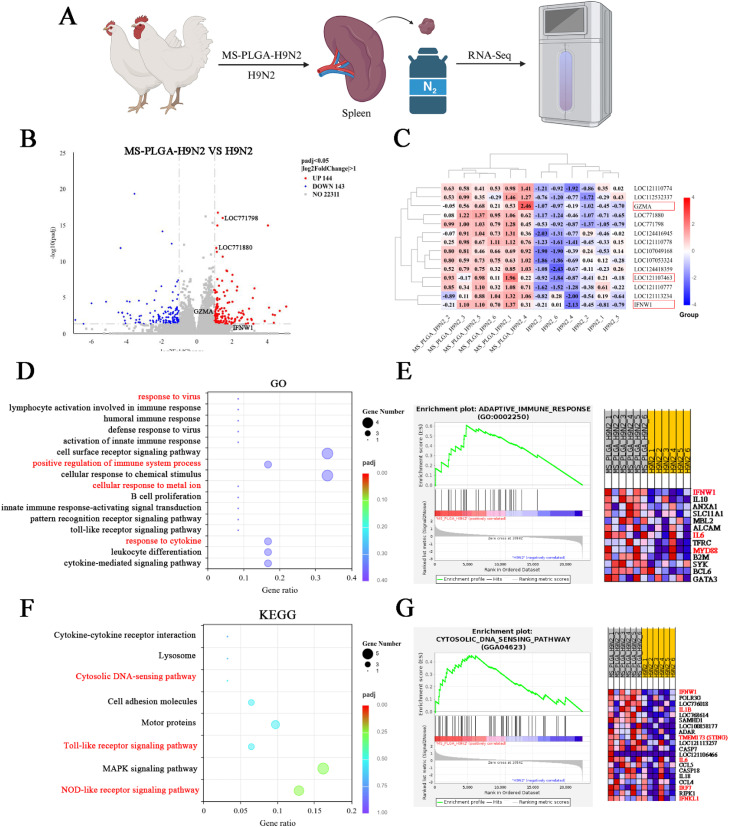
Transcriptomics analysis. (A) Schematic diagram of sample collection. (B) Volcano plot of differentially expressed genes (DEGs). (C) Heatmap of DEGs. (D-E) GO enrichment and GSEA analysis. (F-G) KEGG pathway enrichment and GSEA analysis.

The spleen serves as the primary immune organ in chickens, playing a pivotal role in mounting both humoral and cellular immune responses against foreign antigens. It functions as a critical site for lymphocyte production, maturation, and storage, thereby orchestrating adaptive immunity (Jeurissen, 1993; Smith and Hunt, 2004; Sandford, et al., 2011). To further clarify the impact of MS-PLGA-H9N2 on spleen cell activation, a transcriptomic analysis was performed. The spleen of H9N2 and MS-PLGA-H9N2 groups were collected on the 35th day after first immunization (Fig. 4A). There were 144 up-regulated genes and 143 down-regulated genes between the two groups (Fig. 4B). In selected genes, MS-PLGA-H9N2 substantially upregulated the expression of IFN-β (IFNW1,LOC121107463) and T-cell activation (GZMA,LOC771798) relative genes compare with H9N2 group (Fig. 4C), corroborating its superior immunostimulatory properties, which is consistent with the trend of the above studies. GO analysis, KEGG analysis (Fig. 4DF) and gene set enrichment analysis (GSEA) (Fig. 4EG) further showed that the response induced by MS-PLGA-H9N2 group could positively regulate viral defense and immune response.

### Joint analysis of DEGs/DEPs and immunofluorescence

**Fig. 5:**
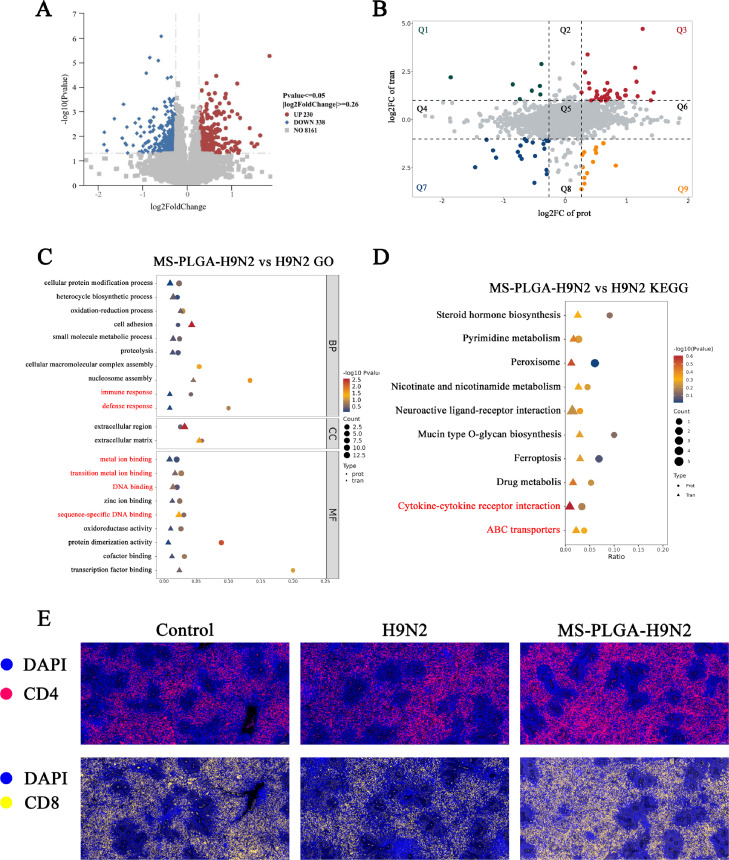
Integrated transcriptomic and proteomic analysis. (A) Volcano plot of differentially expressed proteins (DEPs) between MS-PLGA-H9N2 and H9N2 groups. (B) Nine-quadrant plot of omics correlation analysis. (C) GO pathway enrichment of concordant DEGs/DEPs. (D) KEGG pathway enrichment of concordant DEGs/DEPs. (E) Immunofluorescence of spleen tissue, 10×20, scale bar=50 μm.

It might occur incomplete agreement between biological events described at the gene and protein level due to the influence of MS-PLGA-H9N2 on the spleen. To obtain reliable data, the joint analysis of DEGs and DEPs was employed. There were 8161 differential proteins between MS-PLGA-H9N2 and H9N2, of which 230 proteins were up-regulated and 338 proteins were down-regulated (Fig. 5A). The differential expression analysis showed that 34 factors were significantly up-regulated at the gene and protein levels in the MS-PLGA-H9N2 compared with the H9N2 (Fig. 5B Q3). Moreover, we performed enrichment analysis. First, we explored the functional annotations of DEG/DEPs in the three branches of GO, of which the first 21 GO annotation entries are shown in the Fig. 5C. In the biological (BP) process, DEG/DEP is mainly involved in immune response, defense response and cellular protein modification process. In terms of molecular function (CC), DEG/DEPs are mainly involved in metal ion binding and DNA binding. In terms of cellular localization (MF), DEG/DEPs are mainly involved in the extracellular part. KEGG pathway enrichment analysis revealed the top 10 most significantly enriched pathways based on co-occurrence of both DEGs and DEPs (Fig. 5D). Among them, ABC transporters and cytokine-cytokine receptor interactions were significantly enriched, promoting both antigen processing and lymphocyte activation in immunized chickens. To validate the multi-omics findings, immunofluorescence staining of spleen sections was performed. The results showed that MS-PLGA-H9N2 had stronger activation ability of splenic CD4^+^ and CD8^+^ *T* cells (Fig. 5E), trigger the potent adaptive immune response.

## Discussion

Adjuvants are widely recognized as essential components for most inactivated viral vaccines in poultry (Sasaki and Okuda, 2000). Oil adjuvants remain the most commonly used due to their established safety profile and potent immunogenicity (Cox and Coulter, 1997; Lee, et al., 2014). Oil adjuvant-H9N2 vaccines have been extensively used for decades, however, the epidemiological data reveal that H9N2 continues to circulate worldwide, including recurrent outbreaks among immunized chickens (Lee et al., 2020). This limitation may be attributed to the inability of oil adjuvants to activate T cell-mediated immunity effectively (Reed, et al., 2020), highlighting the urgent need for novel adjuvant formulations. Our study demonstrates that MS-PLGA nanoparticles serve as an effective adjuvant for inactivated H9N2 antigen, significantly enhancing the humoral and cellular immune responses in chickens.

Firstly, we successfully developed PLGA nanoparticles co-encapsulating inactivated H9N2, Mn²⁺, and SMP (MS-PLGA-H9N2). Characterization revealed that the nanoparticles exhibited a mean hydrodynamic diameter of 219.6 ± 5.1 nm and a negative surface charge (−17.7 ± 0.7 mV). MS-PLGA-H9N2 demonstrated excellent stability, maintaining its structural integrity and size characteristics for at least 14 days under 4°C. To evaluate the adjuvant potential of MS-PLGA for H9N2 and compare its efficacy with conventional adjuvants (Algel and ISA), the H9N2 was combined with these adjuvants to immunize chickens by subcutaneous injection in the neck.

Antibody-mediated immunity is crucial for protection against influenza virus infection (Astill, et al., 2018). To evaluate this response, we quantified H9N2-specific IgG antibodies and HI titer at multiple timepoints post-immunization. As shown in Fig. 2CD, the MS-PLGA-H9N2 induced significantly higher antibody titers compared to both Algel-H9N2 and ISA-H9N2 groups, demonstrating its superior capacity to elicit potent humoral immunity. Importantly, on the 35th day after first immunization, the antibody titer remained at a high level, indicating that the sustained release of PLGA effectively prolonged the immune stimulation time and achieved a long-term immune effect.

In addition to humoral immunity, flow cytometry and immunofluorescence analysis showed that MS-PLGA-H9N2 also promoted the activation of peripheral blood lymphocytes and splenic lymphocytes, respectively. It is known that CD4^+^ *T* cells can differentiate into Th1-type cell subsets associated with cellular immune response and Th2-type cell subsets associated with humoral immune response. The two subsets secrete different cytokines to participate in immunity, such as IFN-γ (Th1), which has anti-viral, anti-tumor, and macrophage activation functions; IL-6 (Th2) can promote antibody production (Wusiman, et al., 2022). The results showed that MS-PLGA-H9N2 could significantly upregulate the secretion levels of IFN-γ and IL-6 in serum, suggesting that there was no Th1/Th2 bias. Moreover, we found that IFN-β in serum was also significantly up-regulated by MS-PLGA-H9N2, which further enhanced the antiviral ability of the body. The result was consistent with our previous research (Zhu et al., 2025) and proved that Mn^2+^ carried in MS-PLGA-H9N2 can also promote the secretion of IFN-β in chickens. To assess the biosafety of MS-PLGA-H9N2, we evaluated serum biochemical parameters and histological features at the 35th day. Comparative analysis revealed no significant differences in biochemical indices (ALT, AST, BUN, etc.) or tissue damage (spleen, thymus, bursa of Fabricius) between the MS-PLGA-H9N2 and control groups. These findings demonstrate the excellent biocompatibility of MS-PLGA-H9N2, supporting it as a clinical candidate vaccine.

As the predominant lymphoid organ in chickens, the spleen assumes greater immunological importance compared to mammals due to the underdeveloped lymph node system and limited lymphatic vasculature in avian species (Smith and Hunt, 2004; Sandford er al., 2011). As the primary defense organ against pathogens, it orchestrates both humoral and cellular immune responses. Given this immunological significance, we performed transcriptomic analysis on the spleen from MS-PLGA-H9N2 immunized chickens. Our results demonstrated that MS-PLGA-H9N2 significantly enhanced splenic immune activation compared to H9N2 antigen alone, with the incorporated Mn²⁺ specifically upregulating IFN-β expression, thereby markedly improving chickens antiviral defense capacity. Integrated analysis of GO and KEGG pathways commonly enriched by differentially expressed genes (DEGs) and proteins (DEPs) not only validated the transcriptomic findings but also confirmed that MS-PLGA-H9N2 had a good activation effect on immune response, defense response, cytokine and cytokine receptor interaction related pathways, etc. The results of immunofluorescence also showed that MS-PLGA-H9N2 had stronger activation of splenic CD4^+^ and CD8^+^ *T* cells than the Control and H9N2 groups on the 35th day. Based on the good adjuvant performance of MS-PLGA, we will carry out virus challenge in the future to provide more data support for the clinical application of MS-PLGA.

## Conclusions

In this study, the MS-PLGA-H9N2 nanoparticles were prepared to immunize chickens. Briefly, PLGA encapsulates Mn^2+^, SMP, and inactivated H9N2 to extend their *in vivo* half-life. MS-PLGA-H9N2 triggered stronger humoral and cellular immune responses than H9N2 alone. Owing to the release of Mn^2+^, the concentration of serum IFN-β has been increased significantly, and the analysis of transcriptome and proteomics also supported the above results. Apart from this, the serum biochemical indexes and histological results confirmed that MS-PLGA-H9N2 had good biological safety.

## Ethics statement

This study was performed in strict accordance with the recommendations in the Guide for the Care and Use of Laboratory Animals of the Ministry of Science and Technology of the People's Republic of China. All procedures complied with the Hebei Agriculture University Animal Ethics Committee (NO.2022161).

## Disclosures

The authors declare that they have no known competing financial interests or personal relationships that could have appeared to influence the work reported in this paper.

## CRediT authorship contribution statement

**Yixuan Zhu:** Validation, Investigation, Writing – original draft, Writing – review & editing, Data curation. **Pengfei Gu:** Investigation, Resources. **Yongzhan Bao:** Writing – review & editing. **Bowen Song:** Conceptualization, Methodology, Investigation, Software. **Jinglu Zhang:** Writing – review & editing. **Xiao Wang:** Project administration, Funding acquisition. **Wanyu Shi:** Project administration, Funding acquisition.

## Appendix Supplementary materials

Image, application 1
