# Supplementary material for: Salvia miltiorrhiza polysaccharide-Mn²⁺-PLGA as an adjuvant in H9N2 vaccine: Immunomodulatory effects in chickens
Source: Poult Sci. 2025 Jul 26;104(10):105599. doi: 10.1016/j.psj.2025.105599 (PMC12341616; doi:10.1016/j.psj.2025.105599)
Supplement: Supplementary file 1 [file mmc1.docx]

Short Title: Immunomodulation of MS-PLGA in chickens

***Salvia miltiorrhiza* polysaccharide-Mn²⁺-PLGA as an Adjuvant in H9N2 Vaccine: Immunomodulatory Effects in Chickens**

Yixuan Zhu^a^, Pengfei Gu^a^, Yongzhan Bao^a^, Bowen Song^a^, Jinglu Zhang^a^, Xiao Wang^a,b,*^, Wanyu Shi^a,*^

*^a^ College of Traditional Chinese Veterinary Medicine, Hebei Agricultural University, No. 2596 Lekai South Street, Baoding 071000, China*

*^b^ Hebei Key Laboratory of Traditional Chinese Veterinary Medicine, Baoding, 071001, China*

* *Corresponding author: Hebei Agricultural University, No. 2596 Lekai South Street, Baoding 071000, Hebei, China; E-mail: shiwanyu2010@126.com (W. Shi), wxwangxiao418@163.com (X Wang); Tel: 0086-312-7528355.*


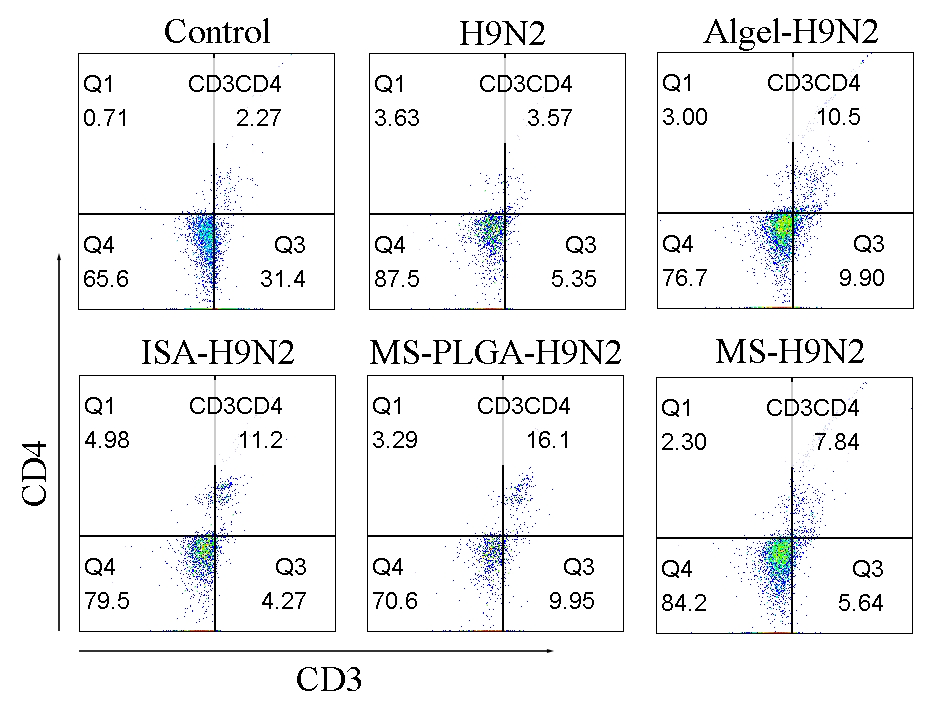


**Fig.S1** Flow scatter plot of CD4^+^ *T* cells


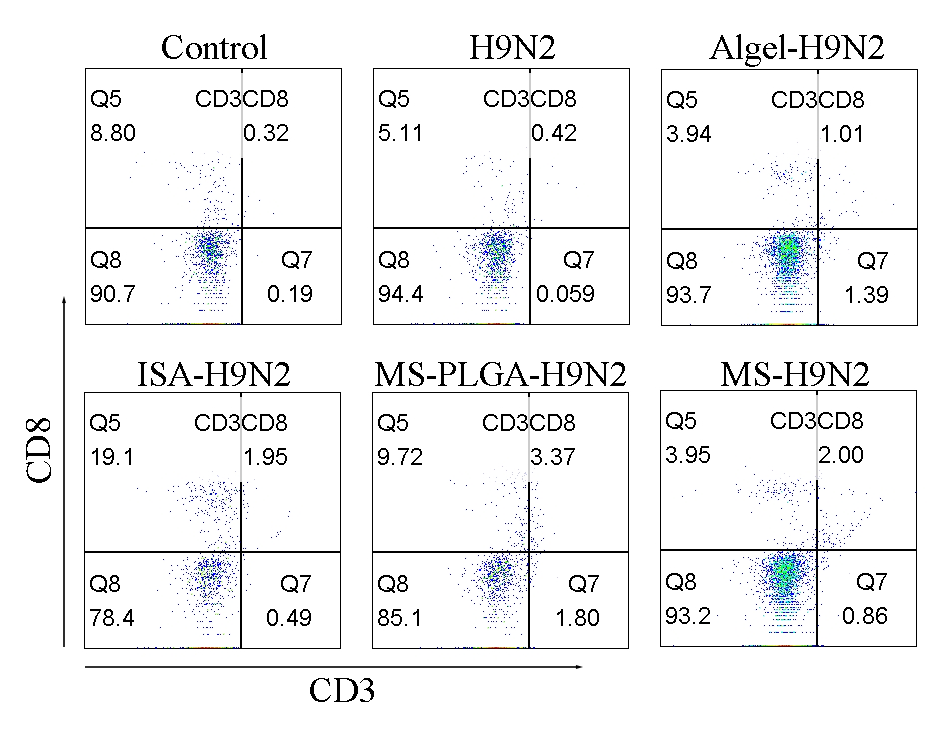


**Fig.S2** Flow scatter plot of CD8^+^ *T* cells


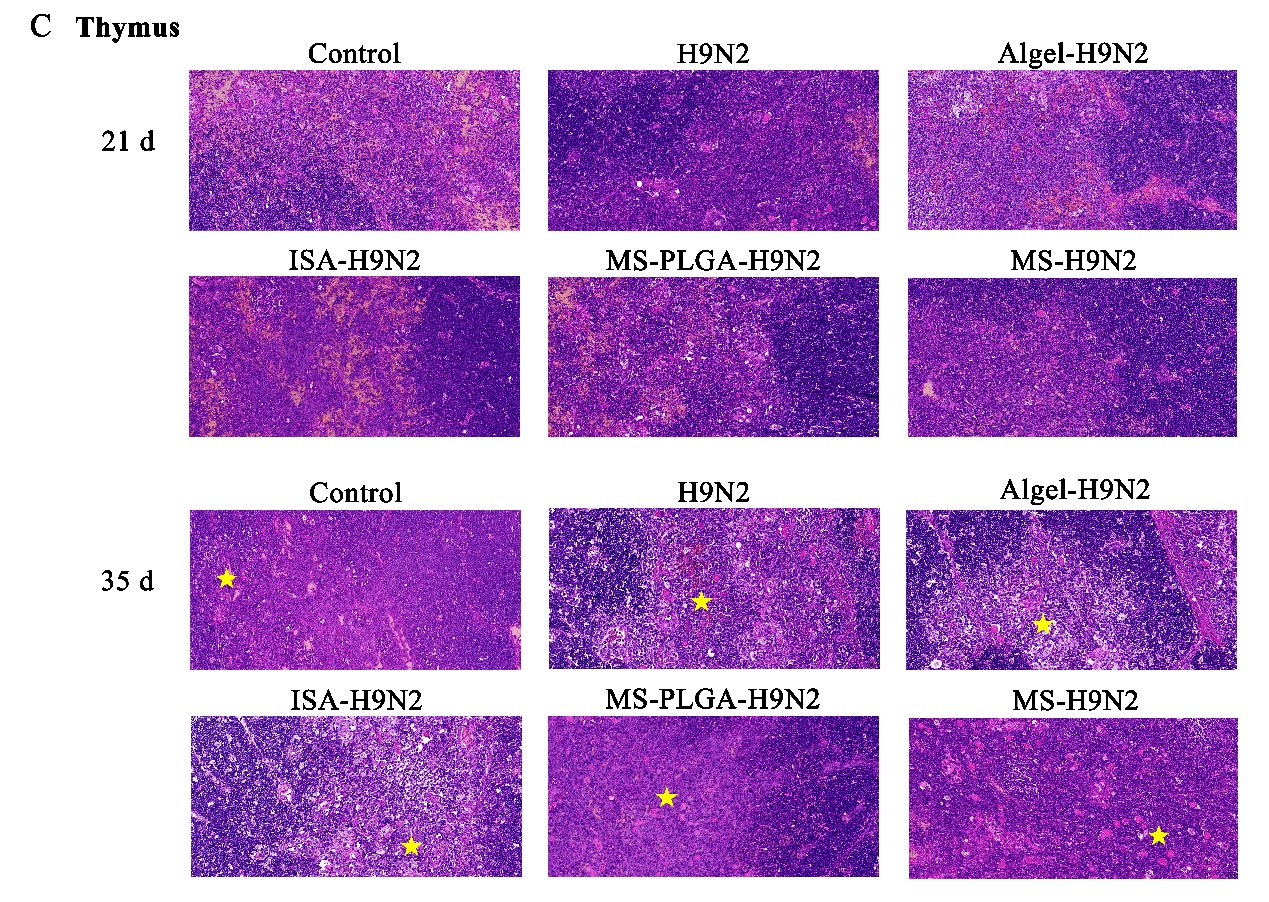


**Fig.S3** Thymic tissue section, ⭐ indicates Hassall's corpuscles, 10×40, scale=20 μm


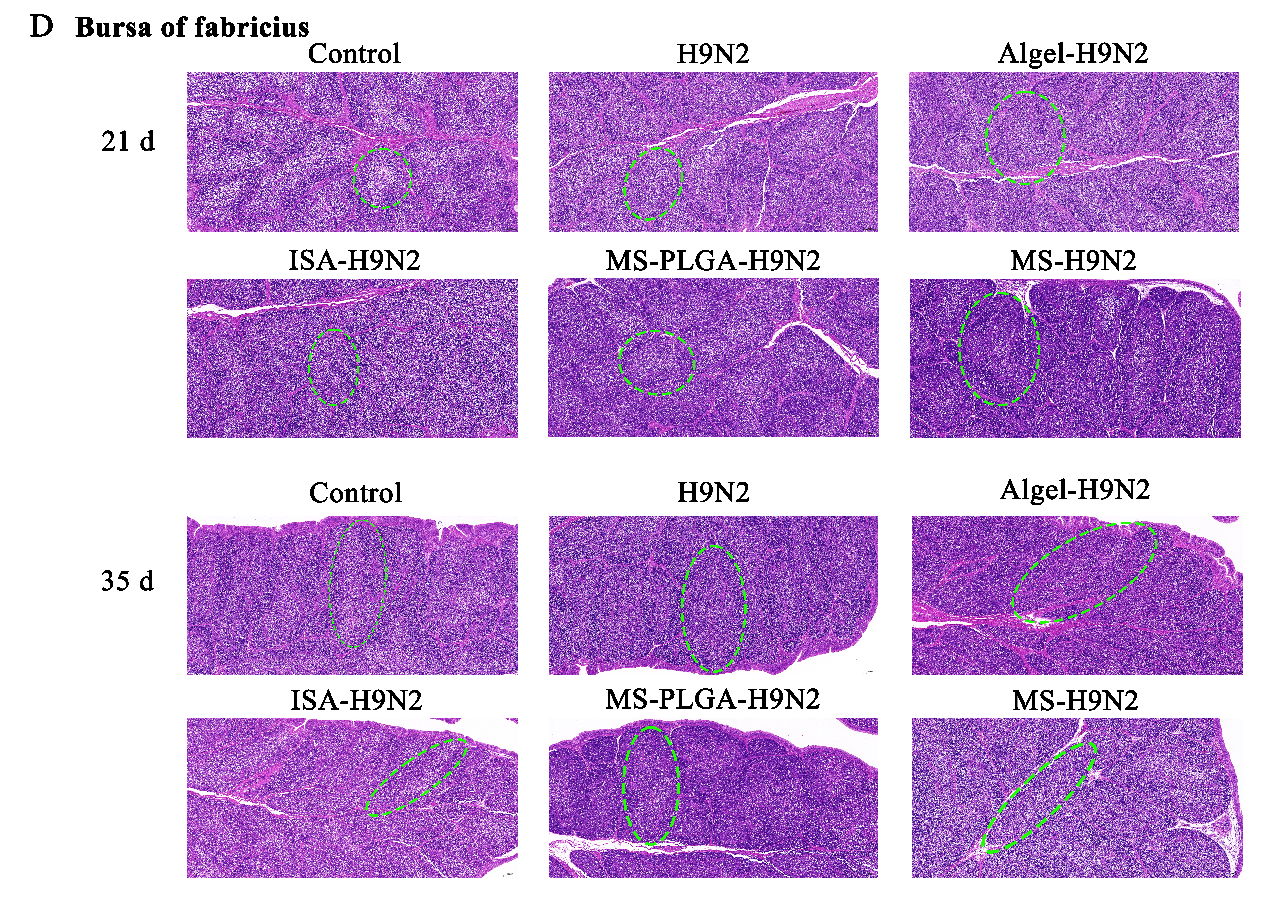


**Fig.S4** Bursa of Fabricius tissue section, green circles highlight lymphoid follicles, 10×10, scale=100 μm
